# Medical oncology workload, workforce census, and needs in Spain: two nationwide studies by the Spanish Society of medical oncology

**DOI:** 10.1007/s12094-023-03225-2

**Published:** 2023-06-15

**Authors:** Ana Fernandez Montes, Elena Elez, Juan de la Haba-Rodriguez, David Paez, Maria Jose Mendez-Vidal, Enriqueta Felip, Alvaro Rodriguez-Lescure

**Affiliations:** 1 Department of Medical Oncology, University Hospital Complex of Ourense (CHUO), Ourense, Galicia Spain; 2 Medical Oncology Department, Vall d’Hebron University Hospital and Institute of Oncology (VHIO), Univesitat Autònoma de Barcelona (UAB), Barcelona, Catalunya Spain; 3 Medical Oncology Department, Reina Sofia University Hospital, Maimonides Institute for Biomedical Research in Cordoba, Córdoba, Spain; 4 Department of Medical Oncology, Santa Creu I Sant Pau University Hospital, U705, ISCIII Centro de Investigación Biomédica en Red de Enfermedades Raras (CIBERER), Barcelona, Catalunya Spain; 5 Medical Oncology Department, Maimonides Institute for Biomedical Research in Cordoba, Reina Sofia University Hospital, Córdoba, Spain; 6 Medical Oncology Department, Vall d’Hebron University Hospital and Institute of Oncology (VHIO), Barcelona, Catalunya Spain; 7 Medical Oncology Department, Elche General University Hospital, Alicante, Spain

**Keywords:** Medical oncologist, Professional standing, Spain, Workforce, Workload, SEOM

## Abstract

**Purpose:**

Growing complexity and demand for cancer care entail increased challenges for Medical Oncology (MO). The Spanish Society of Medical Oncology (SEOM) has promoted studies to provide updated data to estimate the need for medical oncologists in 2040 and to analyse current professional standing of young medical oncologists.

**Methods:**

Two national, online surveys were conducted. The first (2021) targeted 146 Heads of MO Departments, and the second (2022), 775 young medical oncologists who had completed their MO residency between 2014 and 2021. Participants were contacted individually, and data were processed anonymously.

**Results:**

Participation rates reached 78.8% and 48.8%, respectively. The updated data suggest that 87–110 new medical oncologist full-time equivalents (FTEs) should be recruited each year to achieve an optimal ratio of 110–130 new cases per medical oncologist FTE by 2040. The professional standing analysis reveals that 9.1% of medical oncologists trained in Spain do not work in clinical care in the country, with tremendous employment instability (only 15.2% have a permanent contract). A high percentage of young medical oncologists have contemplated career paths other than clinical care (64.5%) or working in other countries (51.7%).

**Conclusions:**

Optimal ratios of medical oncologists must be achieved to tackle the evolution of MO workloads and challenges in comprehensive cancer care. However, the incorporation and permanence of medical oncologists in the national healthcare system in Spain could be compromised by their current sub-optimal professional standing.

## Introduction

Cancer is a public health concern, growing in incidence and prevalence, and a major cause of morbi-mortality worldwide [1, 2]. In Spain, cancer incidence and mortality are estimated to reach 341,450 and 160,271 cases in 2040, respectively [1, 2].

Spain has been a pioneer in establishing the specialty of Medical Oncology [MO], officially recognised in 1978 [3]. The number of positions per year for MO resident interns has currently exceeded 150 in the latest calls [4], with a significant increase in the total number of specialists in training, from 502 in 2014 to 633 in 2021[5].

MO has been defined as a broad, complex specialty, focused on comprehensive cancer patient care and characterised by its academic nature [6]. Medical oncologists not only treat the disease and its complications, but also conduct research and teach, collaborate in the emotional and social support of patients and families and perform important consultancy with other specialties.

MO is thus a demanding and constantly evolving speciality, requiring continuous training and dedication, given the constant inroads being made in cancer therapy [3, 6]. The demands on the specialty have become increasingly onerous, due to the greater incidence of the disease and the ever-changing landscape of clinical care and profile of oncology patients, including aging, lifestyles, increased comorbidity, and longer overall survival with cancer [7]. There has been a paradigm shift in the therapeutic approach and oncology patient care model, with the incorporation of treatment innovations, the promotion of screening and prevention strategies, more complex diagnostic workup, and digital transformation, among others. All these advances have impacted the organisation, activity, and complexity of MO services and have led to subspecialisation as a current trend in modern oncology [8]. It also must be considered that coronavirus 2019 disease (COVID-19) pandemic has brought additional repercussions on the diagnosis and treatment of cancer patients and on professional burnout levels [9, 10].

Despite the escalating demands of the speciality, the MO workforce in Spain remains below the average ratio of oncologists per million population (pmp) in neighbouring countries. In 2018, there were 52 oncologists pmp in Spain, compared to 74 in France, 115 in Germany, 122 in Italy, and 131 in the UK [11].

Within these contexts, the Spanish Society of Medical Oncology (SEOM) conducted two complementary studies to contribute to support the needs of MO staff with updated data, identify corrective actions, and reduce potential risks for the future of MO. The first of these was to update the census of medical oncologists in Spain, obtain solid and up-to-date evidence with respect to the workload of MO and its future evolution, and estimate MO needs on the 2040 horizon. The second was to analyse the current professional standing of young medical oncologists in Spain.

## Methods

*Estimation and evolution of MO workload, census update, and estimated needs for medical oncologists*. An online survey consisting of ninety questions was developed to collect information on the development of the speciality, including current and future estimated workloads. The survey was validated by two study coordinators, both medical oncologists and SEOM board members. The Heads of MO services of the 146 hospitals were invited to participate from January to July 2021.

An analysis of the census of medical oncologists in Spain was calculated in terms of full-time equivalents (FTE), considering an average working week of 40 h.

To determine the number of specialists that will constitute the MO workforce in Spain in 2040, the number of new recruits (trained specialists) and retirements during this time was estimated, assessing training positions published in the Official State Gazette. It was assumed that all new recruits are 26–30 years of age, and the average retirement age was defined as 65 years, although this could be misleading, since 28% of respondents in 2017 planned to retire before that age, and 10% planned to continue working until 67 years of age [12]. Other assumptions were made regarding percentage of trained specialists working in sectors other than clinical care; i.e., medical oncologists who complete their residency and apply for a new speciality or work abroad [12]. The estimated number of medical oncologists needed in Spain in 2040 was calculated according to the projected number of new cancer cases [provided by the Global Cancer Observatory 2020], estimated recruits and retirements, and optimal ratios of medical oncologists per new incident cancer cases/year defined in international studies, in agreement with the study coordinators.

*Professional standing of young medical oncologists in Spain.* To complete specific data*,* a new study was performed to analyse the current professional standing of medical oncologists trained in Spain during 2014–2021. A second online survey was designed, with forty-nine questions to collect data on MO residency, employability, career paths, teaching activities carried out, type of contract, and professional stability. The survey was validated by the SEOM working group set up for this purpose, consisting of four medical oncologists, including SEOM board members, the coordinator of SEOM + MIR Section, and members of its Executive Committee.

A total of 775 young medical oncologists were invited to participate, identified, and contacted based on the information available in the SEOM databases. The survey was open from April to July 2022.

All participants in both studies were informed of the aims of the projects. Completion of the questionnaire was voluntary, and data obtained were treated anonymously. According to the subject nature of the study and data management, approval by an ethics committee was not required.

## Results

### Medical oncologists census update and estimated needs in Spain

A total of 115 questionnaires were analysed (78.8% of the Heads of MO Services invited) with representation from all 17 Autonomous Communities. Eighty-five percent (85%) of the participating hospitals were public; 53% were tertiary or high complexity hospitals; 94% were general hospitals, and 6% monographic cancer centres. This distribution was deemed representative of Spanish hospitals with MO services. On average, there were 164 first consultations per year per medical oncologist, versus 1100–1357 subsequent consultations, with 11.6 medical oncologists per MO Department.

**Fig. 1:**
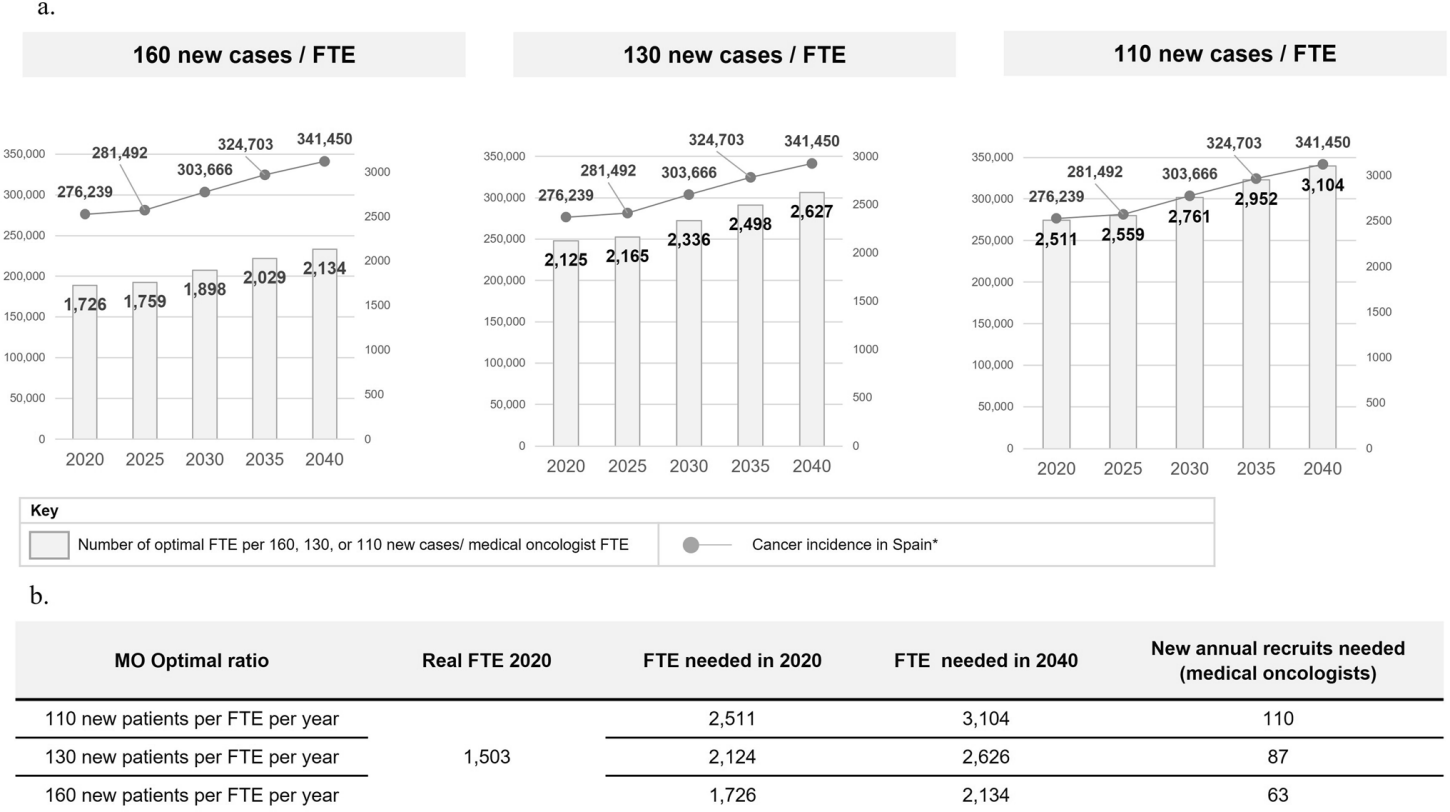
Estimation of needs for medical oncologists 2020–2040, applying optimal ratios. **a** Cancer incidence and number of optimal medical oncologist FTE per 160, 130, or 110 new cases/ medical oncologist FTE, 2020–2040. **b** Number of new annual recruits needed to achieve optimal ratios. Notes: *FTE* Full-time equivalent, *MO* Medical Oncology. Cancer incidence data according to the report, “Cancer rates in Spain 2021”, by the Spanish Society of Medical Oncology (2020: 276.239; 2040: 341.450). *Source: Globocan 2020

Based on the results, it is estimated that there were 1613 medical oncologists working in public and private hospitals in Spain in 2020, corresponding to approximately 1504 FTEs. With these data and current cancer incidence rates, a ratio of 183 new cases/FTE was calculated, being optimal ratios between 130 and 180 [13, 14] or 100 and 150 [15] new cancer cases per medical oncologist FTE, depending on the study. We considered that, given the growing demand and complexity of MO Services in Spain, optimal ratios should target 110–130 new patients per medical oncologist FTE (Fig. 1).

It was estimated that 620 medical oncologists will retire in Spain between 2020 and 2040—almost 40% of the current workforce. Considering this, together with the number of training positions offered and estimated workload evolution (see below), between 87 and 110 new medical oncologists would have to be recruited per year to achieve an optimal ratio of 110–130 new patients per medical oncologist FTE in 2040. In other words, there should be between 2626 and 3104 medical oncologist FTE in Spain; thus, there is an estimated shortage of 1123 and 1601 medical oncologist FTE, respectively (Fig. 1).

### MO workloads: current distribution and estimated changes in 2040

**Fig. 2:**
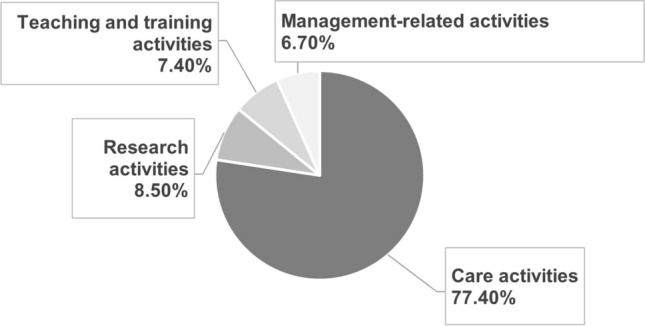
Current distribution of the medical oncologists’ working time

On average, medical oncologists currently dedicate 77.4% of their time to care activities, 8.5% to research, 7.4% to teaching and training, and 6.7% to management-related activities (Fig. 2). A higher percentage of time has been found to be dedicated to research in high complexity hospitals and in MO services with a larger number of beds.

**Fig. 3:**
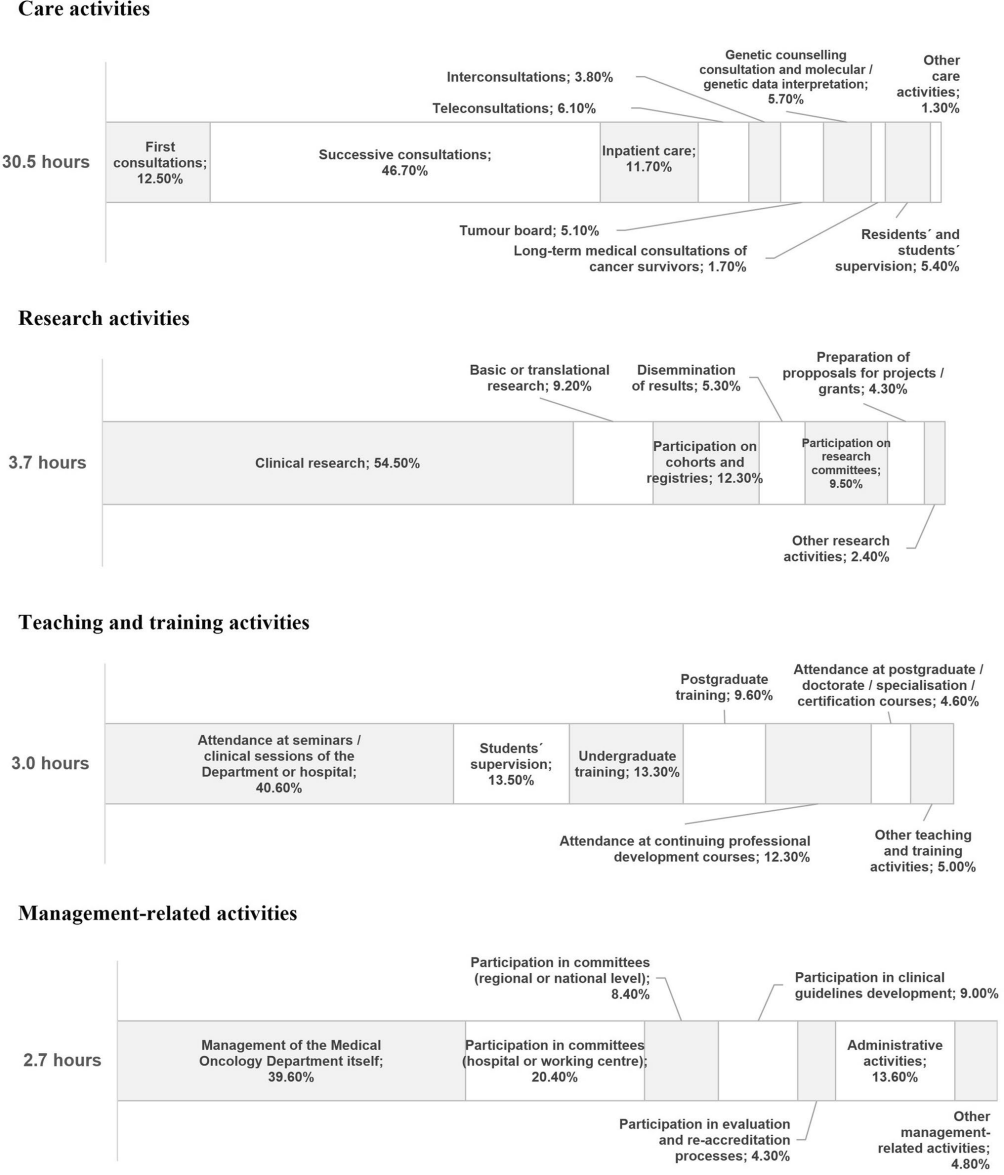
Current distribution of the medical oncologists’ working time by type (care, research, teaching and training, and management activities) and subtype of activity. Notes: Data indicated are expressed as a percentage of the total time spent on each type of activity

Considering 40 h per week, an average of 30.5 h per week are dedicated to care activities; 3 h to teaching and training activities, 3.7 h to research and 2.7 h to management-related activities (Fig. 3). It should be noted that medical oncologists spend 10.9 h per week outside working hours, mainly teaching and research. Taken all together (during their regular workday and outside it), the distribution of their total time is 68% to care activities, 17% to research, 12% to teaching and training, and 8% to management-related activities.

Opinions were collected on how much the time dedicated to each activity will change over the next 20 years (Table 1). Each activity was rated on a scale of 1 (significant decrease) to 7 (significant increase). The results indicate that care and research activities are expected to increase (4.3 points). In primary hospitals, an increase in teaching and training (5.0 points) or management activities (4.5 points) is also expected.

**Table 1:** Medical oncologists’ current dedication and estimation future workload by type of activity in Spain by 2040

Type of activities	Estimated future workload by 2040 (score, 1–7 range)		
	Tertiary hospitals	Secondary hospitals	Primary hospitals
*Care activities*	5.3	5.2	5.3
First consultations	5.1	5.4	5.3
Successive consultations	4.8	4.9	5.3
Inpatient care	4.0	4.0	4.3
Teleconsultations	5.2	5.3	6.7
Interconsultations	4.7	4.8	5.7
Tumour board	5.5	5.3	5.7
Genetic counselling consultation and molecular/ genetic data interpretation	5.9	5.9	6.0
Long-term medical consultations of cancer survivors	4.8	5.0	5.3
Residents’ and students’ supervision^a^	4.9	4.7	5.0
Other care activities	3.6	3.8	5.0
*Research activities*	4.1	3.9	5.0
Clinical research	5.6	5.6	5.3
Basic or translational research	5.5	5.1	4.0
Participation on cohorts and registries	4.6	4.8	5.3
Dissemination of results	4.9	5.0	5.0
Participation on research committees	4.5	4.7	5.0
Preparation of proposals for projects/ grants	5.1	4.8	4.7
Other research activities	3.7	3.7	4.7
*Teaching and training activities*	3.2	2.8	5.0
Attendance at seminars/ clinical sessions of the department or hospital	4.6	4.8	4.3
Student supervision	4.3	4.6	4.0
Undergraduate training	4.4	4.2	3.7
Postgraduate training	4.6	4.5	4.0
Attendance at continuing professional development courses	4.6	4.9	4.7
Attendance at postgraduate/ doctorate/ specialisation/ certification courses	4.4	4.4	4.7
Other teaching and training activities	3.6	3.6	4.3
*Management-related activities*	3.1	2.7	4.5
Management of the Medical Oncology Department itself	4.8	5.1	5.3
Participation on committees (hospital or working centre)	5.0	5.3	5.3
Participation on committees (regionally or nationally)	5.1	5.0	5.3
Participation on developing clinical guidelines	5.0	5.0	5.3
Participation on evaluation and re-accreditation processes	4.9	5.0	5.3
Administrative activities	3.8	4.1	5.0
Other management-related activities	3.0	4.0	5.0

^a^The estimated future workload is indicated as the average of the scores for residents’ supervision activities and students’ supervision activities (clinical field)

### Professional standing of young medical oncologists in Spain

A total of 386 questionnaires were completed in their entirety (participation rate: 49.81%), with representation from all the Autonomous Communities and from all the groups according to the year of finishing the MO residency. 93.0% of the 386 respondents were under the age of 40 years and 63.2% were female. Three specialists did not complete their MO residency in Spain; their answers were therefore not included in the analysis.

The main results of the analysis conducted are indicated in Table 2. The results reveal a high rate of employability for medical oncologists (90.6%), with most of them currently working in Spain (93.21%). Among the 357 specialists trained and working in Spain, 97.5% (348) work in clinical care in MO, either in public or private centres, with a predominance of working only in public hospitals (78.4% work only in public hospitals compared to 8.4% in the private sector). Almost one-third of the specialists surveyed (32.9%) conduct training or teaching activities.

**Table 2:** Professional standing of young medical oncologists in Spain: description of the sample and main results

**Main sample characteristics**	Total	Percentage
**Answers obtained (surveys completed in their entirety)**	**386**	**100%**
*Sex*		
Women	244	63.21%
Men	142	36.79%
*Country of medical oncology residency*		
Spain	383	99.22%
Other countries	3	0.78%
**Professional standing of medical oncologists who did their Medical Oncology Residency in Spain**		
**Medical oncologists trained in Spain**	**383**	**100%**
*Professional standing*		
Employed	347	90.60%
Unemployed	8	2.09%
Leave (temporary, maternity) and leave of absence	16	4.18%
Other professional situations (including completion of a master’s, postgraduate, doctoral degree, fellowship, or other residency)	12	3.13%
*Country of current professional standing*		
Spain	357	93.21%
Other countries	18	4.,70%
Not applicable	8	2.09%
*Teaching activities*		
Involved in any teaching activity	126	32.90%
Master’s level lecturer/ postgraduate studies/ thesis supervision	45	35.71% (out of 126)
Lecturer in continuing professional development/ specialisation courses	38	30.16% (out of 126)
Resident mentoring	34	26.98% (out of)
Other teaching activities (undergraduate training, teaching collaboration with the university, others)	47	37.30% (out of 126)
Is not involved in any teaching activity	257	67.10%
**Activity and professional standing of medical oncologists trained and working in Spain**		
**Medical oncologists trained and working in Spain**	**357**	**100%**
*Area of professional standing*		
Medical Oncology clinical care, including public and/ or private hospitals^a^	348	97.47%
Permanent employment contract	53	15.23% (out of 348)
Temporary employment contract	120	34.48% (out of 348)
Acting official contract	94	27.01% (out of 348)
Research contract (including intensification and R + D + i contracts)	54	15.52% (out of 348)
Other types of contracts (on-call contracts, substitution contract, temporary COVID-19 contract)	26	7.48% (out of 348)
Self-employed	1	0.29% (out of 348)
Pharmaceutical industry	2	0.56%
Research (exclusive)	3	0.84%
Other areas, companies or organisations	4	1.12%
**Stability and professional career. Qualitative aspects of professional activity in Medical Oncology**		
**Medical oncologists trained in Spain**	**383**	**100%**
**Average concern about job stability**	**7.03 / 10**	
*Considering other career opportunities beyond clinical care*		
Yes	64.49%	
No	35.51%	
*Considering working in other countries*		
Yes	51.70%	
No	48.30%	
*Considers their employment situation precarious in terms of Medical Oncology contract*		
Yes	245	63.57%
No	121	31.59%
Not applicable	17	4.44%
*Room for improvement in professional standing of Medical Oncology specialists*		
Yes	378	98.69%
Facilitating access to teaching (average priority, on a scale 1–5)		4.71
Facilitating access to research (average priority, on a scale 1–5)		4.16
Career paths and progression (average priority, on a scale 1–5)		3.49
Workload and number of monthly call-on duties (average priority, on a scale 1–5)		3.42
Remuneration and professional recognition (average priority, on a scale 1–5)		3.33
Stable contracting (average priority, on a scale 1–5)		1.89
No	5	1.31%
*The specialty of Medical Oncology has fulfilled their professional expectations*		
Yes	293	76.50%
No	90	23.50%
*Would choose Medical Oncology again if they had to choose a new speciality*		
Yes	296	77%
No	87	23%

Note ^a^Medical Oncology clinical care includes public hospital exclusively, private hospital exclusively, both (public and private hospital); public hospital and research in public institution, private hospital, and research in private entity and private hospital, and research in public entity

Overall, 9.1% of medical oncologists trained in Spain do not work in oncology care, either because they are unemployed (2.1%), because they work in other countries (4.7%), or in other non-healthcare areas (2.3%), principally the pharmaceutical industry or with exclusive dedication to research.

Only 15.2% of young oncologists working in clinical care in Spain have a permanent position versus 34.5% who have a temporary contract; 27.0% have an acting official contract and 23.3% have another contractual situations, including substitution contracts (4.6%), contracts aimed at intensifying research activity in the national healthcare system (8.9%), research contracts (6.6%), COVID-19 contracts (1.4%), on-call contracts (1.4%), or are self-employed (0.3%). Data indicate greater temporality among females: 13.7% of women working in clinical care in Spain have a permanent employment contract compared to 18.0% of men.

28.7% of the 383 medical oncologists surveyed have signed 5 or more contracts in the last two years. In general, the medical oncologists surveyed identify barriers to access to professional development and improvement; often associated with the type of contract. As for contracts as university professors, 39.3% are associate lecturers and 32.1% assistant lecturers, mostly in the Medical Degree program (96.4%).

Regarding the situation described, 64.0% of the medical oncologists surveyed consider their employment situation to be precarious; 64.5% have considered career paths other than clinical care [mainly working in the pharmaceutical industry or changing clinical care area] and 51.7% have considered working abroad, primarily within the European Union. Among the several reasons alleged, to gain access to better salary conditions (75.6%) and greater professional development (73.1%) were the most frequently cited. 72.9% of the specialists surveyed consider that medical oncologists have worse professional standing in Spain than in other countries.

The average score on concern surrounding job stability was 7.03 out of 10. Overall, 98.7% of the specialists surveyed consider that there is room for improvement in the professional standing of medical oncologists in Spain, largely aimed at facilitating access to teaching and research.

Despite this situation, 76.5% indicated that MO has fulfilled their professional expectations. The main reason expectations have not been met for the remaining 23.5% is the fact that the time for care, research, and training activities is not respected. More than three-quarters of the specialists surveyed (77.3%) would choose MO again if they had to choose speciality.

## Discussion

As part of its commitment to quality healthcare and the future of the specialty, SEOM promoted two studies to define the future needs of MO in Spain in the upcoming years and identify areas for improvement in relation to the workforce and professional standing of medical oncologists in our country.

### MO workloads and trends

According to our data, medical oncologists’ workload was found to be characterised by an imbalance between time dedicated to care activities (77.4% of total time) compared to teaching (7.4%) and research activities (8.5%). It was also revealed that 50% of teaching and training and research activities takes place outside working hours, despite MO being a research-based specialty by definition [6].

Clinical workload of medical oncologists differs significantly between countries in Europe [16] notwithstanding, certain studies define optimal workload standards in MO. The Royal College of Physicians and SEOM have defined that, for a proper balance between the different areas of activity, optimal total care time should be below 72%-75% [6, 12]. For academic and research professional profiles, the percentage of time spent on care activities should be around 55%, with 32% reserved for research work [6]. Dedication to training and teaching activities, having a PhD, and leading research groups may be affected by gender, with a higher proportion of men [17].

Specific time set aside and included within working hours for research and teaching activities are relevant aspects of professional development and part of the appeal for the future leaders in MO [18]. Participants in our study considered facilitating access to research and teaching activities as the two main factors to be addressed to improve their professional standing. But our results have demonstrated that the situation in Spain is far from the defined standards.

A critical look at projected trends in MO clinical practices is a key approach to estimate future needs. Our survey evidence relevant changes in medical oncologists’ projected workload in coming years, with an increase of time dedicated to interpretation of molecular and genetic data and teleconsultation; basic, clinical, and translational research, and to care activities related to increasing complexity of personalised treatments and multidisciplinary care. This evolution of the specialty increases the challenges it faces and requires an innovative approach to workforce planning and training.

### Medical oncologists’ future needs: estimations and potential risks

The possible shortage of oncologists has been reported internationally. In 2014, the American Society of Clinical Oncology estimated that, while the demand for oncology services will increase by 42% in 2025, the number of oncologists will only grow by 28%. This MO shortage was associated with a significant number of anticipated retirements, and the risk of aggravation by high levels of burnout [19]. Similar results have been observed in the specialty of Radiation Oncology (RO) in Spain, with a deficit of 204 radiation oncologists to achieve an optimal ratio of 20 radiation oncologists pmp, bearing in mind the process of technical modernization and increasing demand [20]. In 2017, SEOM developed a plan for MO in Spain that estimated the need for 1 medical oncologist for every 83 new cancer cases per year [12].

Various international studies set the optimal ratios of new cancer cases/medical oncologist FTE as between 130 and 180 [13, 14] or 100–150 for high-income countries [15]. The increase in cancer care demand, accompanied by more complex management and changes in MO workloads, have led us to believe that the optimal ratio of specialists in Spain should tend toward the range of 110–130 new patients/medical oncologist FTE. Theoretically, the annual number of medical oncologists completing MO specialty training (107 in 2020 and 106 in 2021[5]) would make it possible to achieve the number of new recruits needed. But the high number of expected retirements, inadequate professional standing, and the observed imbalance in workloads and burnout are deemed by the authors as major risk factors for maintaining medical oncologists in the national healthcare system.

Our results estimate that almost 40% of the current workforce (620 medical oncologists) will retire between 2020 and 2040, which endorse the need to o ensure generational replacement.

Our survey reveals a high rate of employability for MO, in line with what has been demonstrated [21]. Medical oncologists in Spain typically work in clinical care in the public system [12], this being the main career path contemplated by young medical oncologists and one of the main reasons for choosing MO [22, 23].

Nevertheless, as our data revealed, and in line with other studies, working conditions are far from optimal. Results show high rates of temporality and precarious professional standing for young medical oncologists remaining in Spain and working in healthcare activities, as well as a poor balance between clinical care, teaching, and research, as previously described [12, 21].

According to those surveyed, more than one in three medical oncologists have a temporary contract, with even higher temporality rates among women. Gender inequalities in recruitment and promotion have been identified as a major obstacle to promoting women academics or researchers in biomedical sciences [24]. A study conducted by SEOM in 2021 in Spain revealed that 12.4% of women surveyed were division or unit heads, compared with 45.5% of men, with most women (74.3%) being attending medical oncologists *versus* 45.5% of men [17]. In the RO Spanish workforce analysis, a mere 32% of the specialists in positions of responsibility were women [20]. Encouraging professional development and merit recognition have been identified as key recommendations to bolster the participation and promotion of women in our field of activity [17, 24].

The situation described can increase the risk of burnout, which is especially high in young oncologists and residents [9, 25]. Burnout is associated with several factors, including high demand for care, lack of definition of professional goals and the ever-changing landscape of the healthcare system [25]. Professional well-being and development may well have been further increased by the COVID-19 pandemic [9, 10, 23].

All the aforementioned factors are considered by the authors as a huge obstacle to maintaining and increasing an optimal MO workforce in Spain.

Professional standing and concerns of medical oncologists about stability and professional career have already been addressed in various studies [12, 21, 22, 26], leading them to consider changing their career path. Concern about potential withdrawal from MO clinical care workforce was highlighted by the European Society of Medical Oncology in 2021, by the fact that 25% of the medical oncologist considering changing their future career path [27]. Of them, 38% were contemplating leaving the MO profession, and 28% working in the pharmaceutical industry. In our study, almost one in four respondents expressed the highest concern (score 10/10) for their job stability and 64.0% consider their employment situation to be precarious, due to inadequate contract conditions and the high burden of care activities. This situation has prompted a significant percentage of young medical oncologists trained in Spain (64.5%) to consider other career options as alternatives to clinical care or working in other countries, mainly in Europe, adducing reasons including searching professional development opportunities and better salary conditions, as has been shown [28]. A general concern has been detected in Spain surrounding doctors’ consideration of working abroad: the Spanish General Counsel of Official Medical Colleges issued certificates of competence to an all-time high of 2504 doctors in 2021, most of which were to work in other countries, and mainly for doctors under 36 years [29].

MO speciality has been generally regarded as vocational [30]. Aspects such as the possibilities for promotion and future professional development are the most highly valued factors for choosing MO [30], as well as the transversal nature of this field and its multidisciplinarity [22, 23]. However, high job insecurity, lack of professional development, and high rates of burnout, among other factors, may well compromise underpinning the MO workforce needed to provide adequate training, clinical oncological care, and research in Spain. In our view, actions at multiple levels will be necessary, such as maintaining the number of positions for MO resident interns, guaranteeing an adequate balance between different areas of activity, improving training opportunities, and enhancing employment conditions, including contracts and potential career development.

Both studies performed provide a broad and solid overview of the current and future situation of the MO workforce and activity in Spain. The main limitation of the present survey is a possible selection bias, given the pull effect on the young medical oncologists working in the national public health system who are most often interested in joining SEOM. Bias has been analysed by comparing results obtained in the survey to information available in the SEOM database and those obtained in earlier studies [12, 21].

## Conclusions

The increasing challenges and demand for cancer care, together with the current limited workforce, changes in MO activities, and the need for time set aside for teaching and research, make it advisable to aim for ratios of between 110 and 130 new cancer cases per medical oncologist FTE, leading to the need to incorporate 87–110 new medical oncologists per year into the MO workforce. The number of medical oncologists trained annually in the national health system would theoretically allow these numbers to be reached. However, high number of expected retirements and current sub-optimal professional standing of young medical oncologists, characterised by the lack of professional development, job instability, and an imbalance in the time dedicated to care, research, and teaching activities, may pose a serious risk of brain drain and compromise the incorporation and retention of medical oncologists in the national healthcare system. In this scenario, specific measures are needed to improve the employment situation and workloads of medical oncologists working in clinical practice in Spain and thus maintain quality of care.

## Data Availability

Not applicable.
